# Diabetes: A Risk Factor for Poor Functional Outcome after Total Knee Arthroplasty

**DOI:** 10.1371/journal.pone.0078991

**Published:** 2013-11-13

**Authors:** Jasvinder A. Singh, David G. Lewallen

**Affiliations:** 1 Medicine Service and Center for Surgical Medical Acute care Research and Transitions, VA Medical Center, Birmingham, Alabama, United States of America; 2 Department of Medicine at School of Medicine, and Division of Epidemiology at School of Public Health, University of Alabama, Birmingham, Alabama, United States of America; 3 Department of Orthopedic Surgery, Mayo Clinic College of Medicine, Rochester, Minnesota, United States of America; Charité University Medicine Berlin, Germany

## Abstract

**Background:**

To assess the association of diabetes with postoperative limitation of activities of daily living (ADLs) after primary total knee arthroplasty (TKA).

**Methodology/Principal Findings:**

We used the prospectively collected data from the Mayo Clinic Total Joint Registry to assess the association of diabetes and diabetes with complications with moderate-severe ADL limitation 2- and 5-years after primary TKA. Multivariable logistic regression with general estimating equations adjusted for preoperative ADL limitation, comorbidity and demographic and clinical covariates. Odds ratio (OR) and 95% confidence interval (CI) are presented. 7,139 primary TKAs at 2-years and 4,234 at 5-years constituted the cohorts. In multivariable-adjusted analyses, diabetes was associated with higher odds of moderate-severe limitation at 2- and 5-years, 1.71 (95% CI: 1.26, 2.32; *P = *0.001) and 1.66 (95% CI: 1.13, 2.46; *P = *0.01). Respective ORs for patients with diabetes with complications were 2.73 (95% CI: 1.47, 5.07; *P = *0.001) and 2.73 (95% CI: 1.21, 6.15; *P = *0.016). Sensitivity analyses that adjusted for anxiety and depression or anxiety, depression and ipsilateral hip involvement showed minimal attenuation of magnitude of the association.

**Conclusions/Significance:**

In this large study of patients who underwent primary TKA, diabetes as well as its severity were independently associated with poorer functional outcome. Given the increasing rates of both diabetes as well as arthroplasty, more insight is needed into disease-related and treatment-related factors that underlie this higher risk of ADL limitation in patients with diabetes. Poor functional outcomes may be preventable by modifying the control of diabetes and associated comorbidity in pre- and post-arthroplasty periods.

## Introduction

As the longevity of implants for total knee arthroplasty (TKA) has increased in the recent decades with relatively few failures [1], the focus of arthroplasty outcomes has appropriately shifted to patient-reported outcomes (PROs), including pain, function, quality of life (QOL) and satisfaction [2]. A recent systematic review reported that higher body mass index (BMI), but not age or gender, were associated with functional outcomes after TKA [3]. Four studies including two Canadian, and one English and one U.S. study, with sample sizes ranging from 276 to 7,139 patients, reported that older age, female sex, overall comorbidity, preoperative function, higher BMI were associated with poorer function after TKA [4]–[7]_ Other studies confirmed the association of medical comorbidity with poorer function [8], [9]. However it is not known which common disease/s, predict poorer function after TKA.

Does the functional outcome of primary TKA differ in patients with vs. without diabetes? The answer to this important question is not known. Compared to those without diabetes, patients with diabetes have more complications [10], [11] and a higher risk of revision [12] after TKA. In a recent study of 5,220 primary TKAs, diabetes was associated with differences in functional scores preoperative and up to 7-years postoperative, but pre- and post-operative scores were analyzed together and analyses were not adjusted for important covariates [12]. In contrast, in a study of 222 patients with diabetes and matched controls (age, sex, diagnosis, BMI, length of follow-up, implant design), post-TKA functional scores did not differ between patients with and without diabetes [13]. Thus, it is unclear whether diabetes is associated independently with poorer functional outcomes after primary TKA. Interestingly, two recent studies showed that diabetes is a risk factor for OA and is associated with more severe OA [14], [15]. We hypothesized that diabetes would be independently associated with poorer functional outcomes after primary TKA, patients with diabetes with complications will have even worse functional outcomes and that this association is mediated at least partially by higher comorbidity and a greater number of joints involved in patients with diabetes.

## Methods

We describe the methods and results of our study according to the recommendations from the Strengthening of Reporting in Observational studies in Epidemiology (STROBE) statement [16]. The Institutional Review Board (IRB) at the Mayo Clinic, Rochester, Minnesota, USA approved the study and waived the requirement for informed consent.

### Setting and Participants

Our observational cohort study included all patients who underwent primary TKA at the Mayo Clinic from 1993 to 2005 and had responded to the validated Mayo knee questionnaire [17] at 2- or 5-years after primary TKA.

### Data Sources

Data were obtained from the Mayo Clinic Total Joint Registry, a prospective registry of all patients who undergo joint replacement surgery at the Mayo Clinic, Rochester [1], [18] and linked Mayo Clinic electronic databases. A validated standardized knee questionnaire [17] that assesses knee pain and function (Mayo Knee questionnaire) is administered by mail or during clinic visit preoperatively and at 2- and 5-year time-points post-TKA by experienced, dedicated joint registry staff. The registry staff contact non-responders and those that miss a clinic visit and complete the survey on the phone. Patient-reported outcomes (PROs) have been captured electronically in our Joint Registry since 1993. Several studies using data from the Mayo Knee questionnaire have been published [7], [19]–[22], which is similar to the Knee Society Scale [23].

### Predictor of Interest

The predictors of interest were preoperative diagnoses of diabetes (International Classification of Diseases-ninth revision (ICD-9) codes: 250 – 250.3, 250.7) or diabetes with complications (ICD-9 codes: 250.4 – 250.6), obtained from the Joint Registry and linked electronic databases. We created three mutually exclusive cohorts, controls without diabetes, diabetes without complications and diabetes with complications.

### Covariates

We included several covariates known to be associated with function after TKA [4], [8], [24]-[27] as well as potential confounders, namely: demographics (age, gender); BMI; American Society of Anesthesiologist (ASA) class; implant fixation (uncemented, hybrid/cemented); preoperative ADL limitation; median household income; and distance from the medical center. We also included medical comorbidity assessed using validated Deyo-Charlson index [28], which is a weighted scale of 19 comorbidities (including cardiac, pulmonary, renal, hepatic disease, diabetes, cancer, HIV and so on), expressed as a summative score where a higher score indicates more comorbidity. We also abstracted the presence of anxiety and depression at the time of surgery, based on the presence of ICD-9 codes in the Mayo Clinic electronic databases. Distance from the medical center was included, since patients travel from near and far to Mayo Clinic and the disease severity and patient expectations are likely to differ between these groups, and both can impact arthroplasty outcomes [29].

### Outcomes of Interest

The outcome of interest was overall moderate-severe limitation in ADLs at 2-years or 5-years post-TKA, an undesirable outcome of primary TKA, an elective surgery. Three key ADLs were assessed with the self-reported Mayo Knee questionnaire, including walking, climbing stairs and rising from chair to a standing position via survey, with limitations categorized into ‘no’, ‘mild’, ‘moderate’ or ‘severe’ limitation for each ADL, as previously [7]. Presence of ≥2 activities with moderate or severe limitation was classified *a priori* as overall moderate-severe activity limitation (ref, all other categories), as previously [7], [19]–[22].

### Bias and Sample Size

Since our joint registry includes every patient who underwent TKA at our institution over a 13-year period, we anticipated survey non-response and acknowledge this as a study limitation. We decided *a priori* to compare the characteristics of responders and non-responders to understand the direction of bias and not to impute missing data. We attempted to minimize confounding bias in this cohort study by including the known correlates and confounders of outcomes after TKA including the preoperative functional status. We selected a large enough sample by choosing all eligible patients from 1993–2005 to avoid type II error, i.e., missing the observation of an important finding due to a small sample size. No formal sample size calculations were done, given the lack of previous studies providing effect size estimates. We accounted for the correlation of observations (due to bilateral TKA in a patient, simultaneously or sequential) using appropriate statistical methods.

### Statistical Analyses

We used univariate and multivariable-adjusted logistic regression models, using a generalized estimating equations (GEE) approach, to assess the association between diabetes and moderate-severe ADL limitation 2- and 5-years after primary TKA. This method adjusts for the correlation between observations on the same subject due to both knees having been replaced. The main multivariable model included nine covariates/potential confounders in addition to the predictor of interest, i.e., age, gender, BMI, ASA class, Deyo-Charlson index, operative diagnosis, preoperative ADL limitation, distance from the medical center and income. Sensitivity analyses were done by additionally adjusting the main multivariable model for: (1) anxiety and depression (Model 2); and (2) anxiety, depression and ipsilateral hip involvement (Model 3). Additionally, since the total Deyo-Charlson comorbidity score includes diabetes, we ran the main multivariable model without including Deyo-Charlson index to address the issue of potential collinearity (Model 4). Odds ratios (OR), 95% confidence intervals (CI), and p-values were reported. We compared responder and non-responder characteristics using logistic regression analyses. A p-value <0.05 was considered statistically significant.

## Results

### Clinical and Demographic characteristics and Survey response

The survey response rates were 65% (7,139/10,957) at 2-years and 57% (4,234/7,404 TKAs) at 5-year follow-up. Men, older age and a diagnosis of osteoarthritis were associated with higher likelihood and higher ASA class, higher Deyo-Charlson score and distance of >500 miles from the medical center with lower likelihood of responding to the survey (**Table S1**). There were no differences in response rates between patients with and without diabetes or diabetes with complications (**Table S1**).

The mean age of the 2- and 5-year primary TKA cohorts was 68 and 68 years and mean Deyo-Charlson index score was 1.2 and 1.1, respectively (**Table 1**). For both the 2- and 5-year cohorts, half were women, 18% were 60 years or younger, 7–9% had BMI of 40 or more, 58% had ASA class I-II. 7.1% (509/7,139) and 2.4% (168/7,139) of the 2-year and 8.2% (285/4,234) and 1.6% (68/4,234) of the 5-year cohort had a preoperative diagnosis of diabetes without complications and diabetes with complications, respectively. Compared to patients without diabetes (controls), patients with diabetes with and without complications had higher comorbidity, BMI, income, ASA class and were more likely to be older and have depression, anxiety or ipsilateral hip involvement (**Table S2**).

**Table 1:** Clinical and demographic characteristics of primary TKA cohorts.

	2-year (n = 7,139)	5-year (n = 4,234)
Mean Age (±standard deviation)	68±10	68±10
Men/Women (%)	44%/56%	45%/55%
Age groups (%)		
≤60 yrs	18%	18%
>60–70 yrs	35%	37%
>70–80 yrs	38%	38%
>80 yrs	8%	7%
Body Mass index (%)		
<25 kg/m^2^	13%	13%
25–29.9 kg/m^2^	35%	36%
30–34.9 kg/m^2^	29%	43%
35–39.9 kg/m^2^	14%	7%
≥40 kg/m^2^	9%	7%
American Society of Anesthesiologists (ASA) Class		
Class I-II	58%	58%
Class III-IV	42%	41%
Deyo-Charlson Index, mean (±standard deviation)	1.2±1.9	1.1±1.9
Psychological Comorbidity (%))		
Anxiety	6%	5%
Depression	11%	8%

### Unadjusted and Multivariable-adjusted association of Diabetes with Function after TKA

The unadjusted prevalence of moderate-severe ADL limitation in controls, patients with diabetes without and with complications at each time-point is shown in **Table 2**. Controls, patients with diabetes without complications and patients with diabetes with complications reported moderate-severe ADL limitations as follows: preoperatively, 68.7%, 78.3% and 83.4%; 2-years, 21%, 30.7% and 48.4%; and at 5-years, 27.3%, 44.5% and 64.5% (**Table 2**). Compared to controls, the reduction in moderate-severe ADL limitation was smaller at 2-years in patients with diabetes with complications and at 5-years in both diabetes groups (**Table 2**).

**Table 2:** Unadjusted proportions of patients with moderate-severe ADL limitation preoperatively, 2- and 5-years in the three cohorts

	Control		Diabetes without complications		Diabetes with complications	
	N (%)	Absolute% Reduction (preop –FU period)	N (%)	Absolute% Reduction (preop –FU period)	N (%)	Absolute% Reduction (preop –FU period)
Preoperative mod-severe ADL limitation	4294 (68.7%)		421 (78.3%)		156 (83.4%)	
2-year mod-severe ADL limitation	1281 (21%)	47.7%	147 (30.7%)	57.6%	77 (48.4%)	35.0%
5-year mod-severe ADL limitation	1012 (27.3%)	41.4%	77 (48.4%)	33.8%	40 (64.5%)	18.9%

FU, follow-up

Compared to patients without diabetes, those with diabetes without complications had 1.7- and 2.1-times higher odds of moderate-severe ADL limitation at 2- and 5-years post-TKA (**Table 3**). Respective odds ratios of moderate-severe ADL limitation for patients with diabetes with complications were 3.5 and 4.8, at 2- and 5-years (**Table 3**).

**Table 3:** Univariate and multivariable-adjusted^a^ association of diabetes and diabetes with end-organ damage (complications) with moderate-severe ADL limitation outcomes at 2- and 5-years.

	Univariate				Multivariable-adjusted^a^			
	2-years		5-years		2-years		5-years	
	Odds Ratio (95% CI)	p-value	Odds Ratio (95% CI)	p-value	Odds Ratio (95% CI)	p-value	Odds Ratio (95% CI)	p-value
Control	(reference)		1.0 (reference)		1.0 (reference)		1.0 (reference)	
Diabetes without complications	**1.66 (1.33, 2.07)**	**<0.001**	**2.13 (1.85, 3.13)**	**<0.001**	**1.43 (1.03, 2.00)**	**0.03**	**1.54 (1.01, 2.34)**	**0.04**
Diabetes with complications	**3.52 (2.47, 5.03)**	**<0.001**	**4.83 (2.65, 8.81)**	**<0.001**	**2.96 (1.59, 5.52)**	**0.001**	**2.97 (1.31, 6.71)**	**0.009**

CI, confidence interval; bold represents significant odds ratios

Ref category is the absence of diabetes or diabetes with end-organ damage preoperatively, in the respective models

a Adjusted for 9 additional covariates/confounders: Age, gender, BMI, Deyo-Charlson comorbidity score, ASA class, distance from the medical center, income, underlying diagnosis, preoperative moderate-severe ADL limitation

After adjusting for multiple covariates including socio-demographics, comorbidity and preoperative function, patients with diabetes had 1.4- and 1.5-times higher odds and patients with diabetes with complications had 3.0- and 3.0-times higher odds of moderate-severe ADL limitation at 2- and 5-years post-TKA, respectively (**Table 3** **and Figure S3**).

Sensitivity analyses that additionally adjusted for anxiety and depression (model 2) or anxiety, depression and ipsilateral hip involvement (model 3) or excluded Deyo-Charlson index from the main model due to potential concern for collinearity (model 4) showed minimal attenuation of odds ratios (**Table 4**).

**Table 4:** Sensitivity analyses of multivariable-adjusted association of diabetes with moderate-severe ADL limitation at 2- and 5-years

	2-years		5-years	
	OR (95% CI)	p-value	OR (95% CI)	p-value
Model 2 (Model 1 +anxiety + depression)^a^				
**Control**	1.0 (ref)		1.0 (ref)	
**Diabetes without complications**	**1.45 (1.04, 2.01)**	**0.03**	**1.59 (1.04, 2.41)**	**0.03**
**Diabetes with complications**	**2.49 (1.59, 5.47)**	**0.001**	**3.00 (1.28, 7.06)**	**0.01**
Model 3 (Model 1 +anxiety + depression + ipsilateral hip involvement)^b^				
**Control**	1.0 (ref)		1.0 (ref)	
**Diabetes without complications**	1.37 (0.92, 2.06)	0.12	1.46 (0.87, 2.45)	0.15
**Diabetes with complications**	**2.51 (1.23, 5.09)**	**0.01**	**3.13 (1.29, 7.61)**	**0.01**
Model 4 (Model 1 minus Deyo-Charlson index)^c^				
**Control**	1.0 (ref)		1.0 (ref)	
**Diabetes without complications**	**1.56 (1.14, 2.17)**	**0.006**	**1.60 (1.51, 2.43)**	**0.03**
**Diabetes with complications**	**3.86 (2.13, 6.99)**	**<0.001**	**3.34 (1.51, 7.37)**	**0.003**

a Adjusted the main multivariable model for anxiety and depression, i.e. the model is adjusted for a total of 11 additional covariates/confounders: Age, gender, BMI, Deyo-Charlson comorbidity score, ASA class, distance from the medical center, income, underlying diagnosis, preoperative moderate-severe ADL limitation, anxiety and depression

b Adjusted the main multivariable model for anxiety, depression and ipsilateral hip pain, i.e. the model is adjusted for a total of 12 additional covariates/confounders: Age, gender, BMI, Deyo-Charlson comorbidity score, ASA class, distance from the medical center, income, underlying diagnosis, preoperative moderate-severe ADL limitation, anxiety, depression and ipsilateral hip involvement

c Adjusted the main multivariable model for anxiety and depression, but not for Deyo-Charlson, i.e. the model is adjusted for a total of 8 additional covariates/confounders: Age, gender, BMI, ASA class, distance from the medical center, income, underlying diagnosis, preoperative moderate-severe ADL limitation

## Discussion

In this study, we examined a preoperative diagnosis of diabetes as a potential independent predictor of functional outcomes at 2- and 5-years after primary TKA in large cohorts of patients at each time point, using an institutional total joint registry that collects data prospectively with the help of dedicated joint registry staff. We found that diabetes was significantly independently associated with poor functional outcome at both 2- and 5-years after primary TKA. The association was noted even after adjustment for several factors including preoperative functional limitation. Our findings are robust and merit further discussion.

Our main finding was that diabetes increased the odds of moderate-severe ADL limitation in patients undergoing primary TKA by almost 2-fold compared to patients without diabetes. This is a significant finding and adds to the current knowledge in this field. In a recent study of 5,220 primary TKAs using unadjusted analyses, diabetes was associated with overall differences in functional scores that included pre- and post-operative scores analyzed together [12]. Since preoperative functional scores have been noted to be lower in patients with diabetes,[12], [30] it wasn't clear whether lower post-operative functional scores in patients with diabetes in this study were due to preoperative differences or due to diabetes. In contrast, a study of 222 patients with diabetes with controls matched (1∶1) for age, sex, diagnosis, BMI, length of follow-up and implant design, found no differences in either preoperative or postoperative functional scores at mean follow-up of 4.5 years [13]. Both studies used the same outcome instrument (knee society score), but differed in that the positive study did not adjust for any factor, while the negative study was adjusted for several factors. Thus, the positive study findings seemed confounded, due to the lack of adjustment for important covariates. Post-operative scores were not adjusted for preoperative differences or comorbidities in either study.

Our study is the first to adjust for both comorbidity and preoperative functional limitation. These are important factors to adjust in analyses, given that they are strongly associated with post-TKA function outcomes [4]-[9]. In addition, as evident in appendices 2-3, these and several other covariates adjusted in our study also differed significantly between patients with and without diabetes, highlighting the importance of including them in the multivariable-adjusted analyses. Thus, our study provides robust data and adds clarity. Our main finding was that patients with diabetes have worse functional outcomes than those without diabetes after primary TKA even after controlling for differences in preoperative functional limitation, a strong predictor of post-arthroplasty functional outcomes. So, what leads to poor functional outcomes in patients with diabetes? Several potential reasons may contribute.

Diabetes has been shown to be an independent risk factor for ADL limitation in population-based studies in the general population [31], [32]; the same risk factors and pathways to poor functional status in patients with diabetes in the general population may also extend to post-TKA period. Patients with diabetes have higher risk of post-surgical complications, [10], [11] including deep infection rate [33]–[35] and postoperative neuropathy[30], that may interfere with recovery from TKA and likely contribute to suboptimal functional limitation. As we hypothesized, adjustment for comorbidity (main model) led to attenuation of association of diabetes with ADL limitation, thus indicating this association was mediated, at least partially, by medical comorbidity in patients with diabetes. A higher comorbidity in patients with diabetes may increase the risk of post-operative complications including infections and neuropathy and may impact the functional recovery from this major surgery in a variety of ways, leading to more ADL limitations. Recent studies have also shown that diabetes is independently associated with risk of OA and more severe OA,[14], [15]. Thus, patients with diabetes would be hypothesized to have a more extensive OA involvement (i.e. ipsilateral hip involvement). In fact, ipsilateral hip involvement seemed to partially mediate higher ADL limitation in patients with diabetes in our study (sensitivity analyses), confirming that more severe OA in patients with diabetes is an important determinant of higher ADL limitation after primary TKA. Interestingly, psychological comorbidity seemed not to mediate the higher ADL limitations in patients with diabetes post-TKA, as evident by no significant change in odds ratios when adjusting the main multivariable-adjusted model additionally for anxiety and depression. This also adds to the current knowledge.

Complications such as retinopathy, nephropathy, neuropathy and myopathy associated with diabetes indicate a longer disease duration and/or more severe disease. These complications would be expected to interfere with physical rehabilitation after primary TKA, which is associated with the long-term outcomes after primary TKA [36]. This may explain the why the functional outcomes seen in patients with diabetes with complications were the worst, and even higher than in patients with diabetes alone. Our observation of higher ADL limitation after primary TKA in patients with diabetes with complications is similar to those in a population study, [32] that noted that complications as well as the longer diabetes disease duration were associated with more ADL limitation [32]. To our knowledge, our study is the first to establish this finding in post-TKA population.

Several facts support the robustness of our study findings. First, the effect estimates were minimally attenuated between unadjusted and the main multivariable-adjusted model. Second, the multivariable-adjusted estimates changed minimally after adjustment for additional covariates in sensitivity analyses. Third, the estimates were stable across the 2- and 5-year time-points. Fourth and most important, we noted a dose effect with odds being higher in patients with diabetes compared to patients without diabetes and even higher in patients with diabetes with end-organ damage. The increasing odds of poor functional outcome from patients without diabetes, with diabetes and with diabetes with complications confirm a dose-effect, a key criteria for causality. This finding also supported our hypothesis that diabetes and its severity were associated with poorer functional outcome after primary TKA. Thus, our study provides evidence of temporal relationship, strength of association, dose-response relationship, consistency, plausibility, specificity and coherence, meeting most of Bradford Hill's criteria of causation [37].

Our study findings must be interpreted considering study limitations. The combination of various modes of administration may have added variability, although a very high correlation has been demonstrated between mailed and in-person physician-administered Mayo Knee questionnaire [17]. This was a single center study, which might raise the question of generalizability of our findings to other settings. However, the demographic characteristics of our study cohort are similar to those in the previously published studies of U.S. cohorts [38], [39], confirming the representativeness of this sample. This is not surprising since the medical center provides health care to the local community as well as serves as a referral base. Non-response may have biased our findings, especially for the 5-year follow-up; however, the response rate is in the range of 60% reported for large surveys [40]. Our analyses were not adjusted for severity of OA in other joints, but we aimed to account at least partially for OA in other lower extremity joints by including the variable ipsilateral hip involvement, a large joint associated with significant morbidity and a suspected contributor to lower extremity functional limitation. Functional improvement after TKA is not the only goal; pain relief is the other important goal, which was not evaluated in this study.

## Conclusions

In conclusion, in this study we found a strong association of a preoperative diagnosis of diabetes with postoperative ADL limitation 2- and 5-years after primary TKA. The association was partially mediated by medical comorbidity and several demographic differences between patients with and without diabetes. Patients with more severe diabetes i.e. diabetes with complications similarly had an even stronger association with ADL limitation 2- and 5-years after primary TKA. These findings should be used to inform the discussion with patients with diabetes during the informed consent process, so that these patients are aware of expected outcomes after primary TKA. Studies are also needed to examine if pre- and post-operative management of medical or arthritic comorbidity in patients with diabetes undergoing primary TKA can improve the functional outcomes after primary TKA in diabetic patients.

## Supporting Information

Figure S1 Whiskers represent 95% confidence intervals DM, Diabetes Mellitus
(DOCX)

Table S1 ^*^P<0.05; ^‡^ p<0.01, ^†^p<0.001 All other p-values are ≥0.05, unless indicated as above
(DOCX)

Table S2 BMI, body mass index: ASA, American Society of Anesthesiologists; RA; rheumatoid arthritis
(DOCX)
