# Supplementary material for: Diabetes: A Risk Factor for Poor Functional Outcome after Total Knee Arthroplasty
Source: PLoS One. 2013 Nov 13;8(11):e78991. doi: 10.1371/journal.pone.0078991 (PMC3827297; doi:10.1371/journal.pone.0078991)

**Supporting Information**

**Figure S3. Multivariable-adjusted association of diabetes with moderate-severe ADL limitation at 2- and 5-years post-TKA (Main Model)**


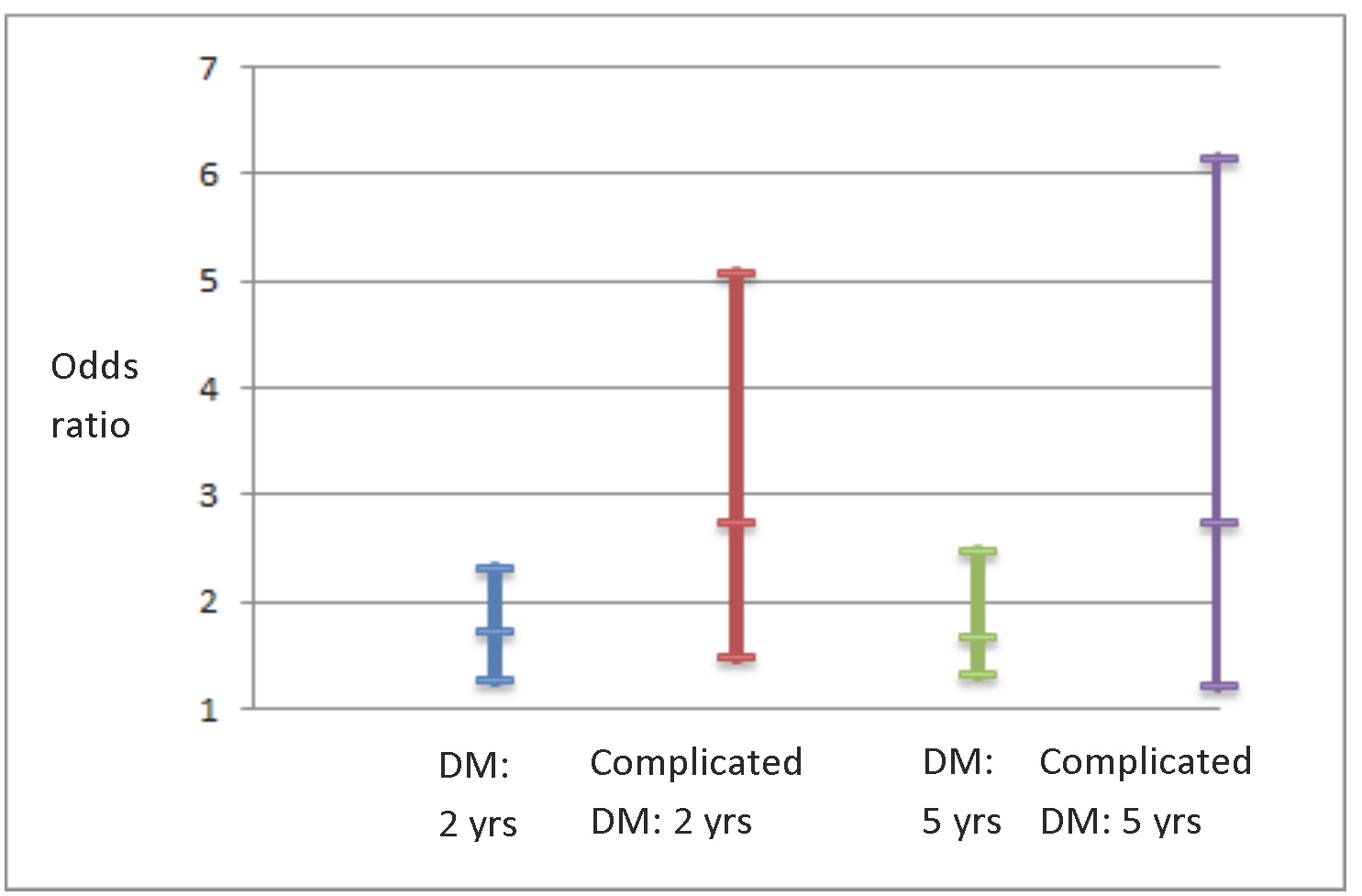

Supplement: Figure S1 — Whiskers represent 95% confidence intervals DM, Diabetes Mellitus (DOCX) [file pone.0078991.s001.docx]
