# Supplementary material for: Diabetes: A Risk Factor for Poor Functional Outcome after Total Knee Arthroplasty
Source: PLoS One. 2013 Nov 13;8(11):e78991. doi: 10.1371/journal.pone.0078991 (PMC3827297; doi:10.1371/journal.pone.0078991)
Supplement: Table S1 — *P<0.05; ‡ p<0.01, †p<0.001 All other p-values are ≥0.05, unless indicated as above (DOCX) [file pone.0078991.s002.docx]

**Supporting Information**

**Table S1. Non-responder characteristics**

|  | **2-yr primary TKA** | | **5-yr primary TKA** | |
| --- | --- | --- | --- | --- |
|  | **Events for non-responders**  **(n/N= 3818/**  **10957)** | **Odds Ratio**  **(95 % Confidence Interval)** | **Events for non-responders (n/N= 3170/7404)** | **Odds Ratio**  **(95 % Confidence Interval)** |
| **Gender** |  |  |  |  |
| Women | 2184/6161 (35.4%) |  | 1860/4191 (44.4%) |  |
| Men | 1634/4796 (34.1%) | 0.94  (0.86,1.03) | 1310/3213 (40.8%) | 0.86 ‡ (0.78,0.96) |
| **Age groups n (%)** |  |  |  |  |
| ≤60 yrs | 841/2154 (39%) |  | 728/1473 (49.4%) |  |
| >60-70 yrs | 1273/3804 (33.5%) | 0.79 ^‡^  (0.69,0.89) | 1065/2641 (40.3%) | 0.69 ^‡^ (0.60,0.80) |
| >70-80 yrs | 1387/4121 (33.7%) | 0.79 ^‡^ (0.70,0.89) | 1142/2759 (41.4%) | 0.72 ^‡^ (0.63,0.83) |
| >80 yrs | 317/878 (36.1%) | 0.88 (0.74,1.05) | 235/531 (44.3%) | 0.81 (0.65,1.01) |
| **BMI Categorized** |  |  |  |  |
| ≤24.9 | 514/1474 (34.9%) |  | 452/1018 (44.4%) |  |
| 25-29.9 | 1287/3766 (34.2%) | 0.97 (0.84,1.11) | 1061/2586 (41%) | 0.87 (0.74,1.02) |
| 30-39.9 | 1644/4712  (34.9%) | 1.00 (0.87,1.15) | 1346/3169 (42.5%) | 0.92 (0.79,1.08) |
| ≥40 | 355/960 (37%) | 1.10 (0.91,1.32) | 299/602 (49.7%) | 1.24 (0.99,1.55) |
| **ASA** |  |  |  |  |
| 1-2 | 2021/6136 (32.9%) |  | 1771/4238 (41.8%) |  |
| 3-4 | 1772/4778 (37.1%) | 1.20 ^‡^ (1.10,1.31) | 1388/3129 (44.4%) | 1.11 * (1.00,1.23) |
| **Deyo-Charlson index (5 point increase)** |  | 1.30 ^‡^ (1.17,1.44) |  | 1.07 (0.93,1.22) |
|  |  |  |  |  |
| Annual household Income |  |  |  |  |
| ≤$35,000 | 1035/3099 (33.4%) |  | 720/1665 (43.2%) |  |
| >$35,000 to $45,000 | 699/2098 (33.3%) | 1.00 (0.87,1.14) | 736/1841 (40%) | 0.87 (0.75,1.02) |
| >$45,000 | 1347/4044 (33.3%) | 1.00 (0.89,1.11) | 1058/2541 (41.6%) | 0.94 (0.82,1.07) |
| **Distance** |  |  |  |  |
| 0-100 miles | 1785/5454 (32.7%) |  | 1443/3523 (41%) |  |
| >100-500 miles | 1435/4166 (34.4%) | 1.08 (0.98,1.19) | 1218/2871 (42.4%) | 1.06 (0.95,1.19) |
| >500 miles or  Non-US | 476/1017 (46.8%) | 1.81 ^‡^ (1.55,2.11) | 382/709 (53.9%) | 1.68 ^‡^ (1.40,2.03) |
| **Underlying Diagnoses** |  |  |  |  |
| Inflammatory Arthritis | 172/428 (40.2%) |  | 155/344 (45.1%) |  |
| Osteoarthritis | 3480/10190 (34.2%) | 0.77 * (0.62,0.97) | 2872/6794 (42.3%) | 0.89 (0.70,1.14) |
| Other | 166/338 (49.1%) | 1.44 * (1.05,1.96) | 143/266 (53.8%) | 1.42 * (1.00,2.01) |
| **Diabetes** |  |  |  |  |
| No | 4260/9855 (43.2%) |  | 2687/6732 (39.9%) |  |
| Yes | 441/1099  (40.1%) | 0.9  (0.8,1.0) | 248/672  (36.9%) | 0.9  (0.7,1.1) |
| **Diabetes with complications** |  |  |  |  |
| No | 4590/10658 (43.1% |  | 2885/7245 (39.8% |  |
| Yes | 111/296  (37.5%) | 0.8  (0.6,1.0) | 50/159  (31.4%) | 0.7  (0.5,1.0) |
