# Supplementary material for: Diabetes: A Risk Factor for Poor Functional Outcome after Total Knee Arthroplasty
Source: PLoS One. 2013 Nov 13;8(11):e78991. doi: 10.1371/journal.pone.0078991 (PMC3827297; doi:10.1371/journal.pone.0078991)
Supplement: Table S2 — BMI, body mass index: ASA, American Society of Anesthesiologists; RA; rheumatoid arthritis (DOCX) [file pone.0078991.s003.docx]

**Supporting Information**

Table S**2.** Clinical and demographic characteristics of patients with diabetes, diabetes with complication and controls

|  | **2-year** | | | | **5-year** | | | |
| --- | --- | --- | --- | --- | --- | --- | --- | --- |
|  | **Controls** | **Diabetes** | **Complicated Diabetes** | **p-value** | **Controls** | **Diabetes** | **Complicated Diabetes** | **p-value** |
| Mean Age (±standard deviation) | 68.3±10.1 | 69.3±8.1 | 69.5±8.6 | 0.035 | 68.4±9.7 | 69.1±7.7 | 68.2±9.7 | 0.48 |
| Mean BMI | 30.6±5.8 | 34.4±6.6 | 34.7±7.7 | <0.001 | 30.5±5.6 | 34.1±6.5 | 34.8±8.5 | <0.001 |
| Deyo-Charlson Index, mean (±SD) | 1.0±1.8 | 2.4±2.0 | 5.2±2.4 | <0.001 | 1.0±1.8 | 2.3±1.9 | 5.0±2.4 | <0.001 |
| % female | 56.2% | 52.1% | 47.0% | 0.014 | 55.4% | 50.5% | 51.5% | 0.23 |
| Age groups (%) |  |  |  | 0.002 |  |  |  | 0.04 |
| ≤60 yrs | 19.0% | 13.0% | 12.5% |  | 17.8% | 14.7% | 16.2% |  |
| >60-70 yrs | 34.9% | 40.7% | 40.5% |  | 37.1% | 37.8% | 42.6% |  |
| >70-80 yrs | 38.1% | 40.3% | 39.3% |  | 37.8% | 44.6% | 35.3% |  |
| >80 yrs | 8.0% | 6.1% | 7.7% |  | 7.3% | 2.8% | 5.9% |  |
| Body Mass index (%) |  |  |  | <0.001 |  |  |  | <0.001 |
| <25 kg/m^2^ | 14.3% | 4.6% | 8.3% |  | 14.2% | 3.9% | 8.8% |  |
| 25-29.9 kg/m^2^ | 36.2% | 23.0% | 19.0% |  | 37.5% | 22.5% | 17.6% |  |
| 30-34.9 kg/m^2^ | 29.1% | 32.7% | 31.0% |  | 28.9% | 38.4% | 35.3% |  |
| 35-39.9 kg/m^2^ | 13.2% | 19.4% | 17.3% |  | 13.2% | 18.7% | 11.8% |  |
| ≥40 kg/m^2^ |  |  |  |  | 6.2% | 16.5% | 26.5% |  |
| ASA Class |  |  |  | <0.001 |  |  |  | <0.001 |
| Class I-II | 60.3% | 37,1% | 25.0% |  | 60.8% | 38.2% | 19.1% |  |
| Class III-IV | 39.7% | 62.9% | 75.0% |  | 39.2% | 61.8% | 80.9% |  |
| Psychological Comorbidity (%)) |  |  |  |  |  |  |  |  |
| Anxiety | 6.3% | 7.5% | 11.9% | 0.01 | 5.1% | 5.6% | 10.3% | 0.15 |
| Depression | 9.9% | 11.8% | 23.2% | <0.001 | 7.8% | 8.8% | 10.3% | 0.64 |
| Ipsilateral hip involvement | 11.8% | 13.7% | 20.7% | 0.01 | 13.2% | 15.9% | 14.6% | 0.53 |
| Annual household Income |  |  |  | <0.001 |  |  |  | 0.28 |
| ≤$35,000 | 23.5% | 17.1% | 10.0% |  | 31.7% | 28.6% | 20.3% |  |
| >$35,000 to $45,000 | 43.8% | 45.5% | 37.3% |  | 41.8% | 43.2% | 45.4% |  |
| >$45,000 | 32.7% | 37.4% | 52.7% |  | 26.5% | 28.2% | 34.4% |  |
| Distance from Medical Center |  |  |  | <0.012 |  |  |  | 0.19 |
| 0-100 miles | 52.3% | 56.5% | 63.3% |  | 51.0% | 51.8% | 63.1% |  |
| >100-500 miles | 39.7% | 37.9% | 31.9% |  | 40.7% | 42.0% | 33.8% |  |
| >500 miles | 8.0% | 5.6% | 4.8% |  | 8.3% | 6.2% | 3.1% |  |
| % cemented/hybrid implant | 98.1% | 98.6% | 98.8% | 0.57 | 99.7% | 98.6% | 100% | 0.89 |
| Operative diagnosis |  |  |  | 0.032 |  |  |  | 0.19 |
| RA/inflammatory arthritis | 3.8% | 1.6% | 1.8% |  | 4.6% | 2.1% | 4.4% |  |
| Osteoarthritis | 93.7% | 96.9% | 96.4% |  | 92.3% | 96.1% | 94.1% |  |
| Other | 2.5% | 1.6% | 1.8% |  | 3.0% | 1.8% | 1.5% |  |
